# Hybrid Pennisetum colonization by *Bacillus megaterium* BM18-2 labeled with green fluorescent protein (GFP) under Cd stress

**DOI:** 10.1007/s00203-024-04228-5

**Published:** 2025-01-09

**Authors:** Nehal Kamal, Chen Qian, Huanhuan Hao, Juanzi Wu, Zhiwei Liu, Xiaoxian Zhong, Osama M. Ghanem, Ali Salem, Zoltan Orban, Abdallah Elshawadfy Elwakeel, Samy F. Mahmoud, Alaa F. Said

**Affiliations:** 1 National Forage Breeding Innovation Base (JAAS), Nanjing, 210014 People’s Republic of China; 2 Institute of Animal Science, Jiangsu Academy of Agricultural Sciences, Nanjing, 210014 People’s Republic of China; 3 Key Laboratory for Crop and Animal Integrated Farming of Ministry of Agriculture and Rural Affairs, Nanjing, 210014 People’s Republic of China; 4 Department of Botany and Microbiology, Faculty of Science, Suez University, P.O. Box: 43221, Suez, Egypt; 5 College of Agro-grassland Science, Nanjing Agricultural University, 210095 Nanjing, People’s Republic of China; 6 Soil and Water Department, Faculty of Agriculture, Suez Canal University, 41522 Ismailia, Egypt; 7 Civil Engineering Department, Faculty of Engineering, Minia University, Minya, Egypt; 8 Structural Diagnostics and Analysis Research Group, Faculty of Engineering and Information Technology, University of Pécs, Pécs, Hungary; 9 Agricultural Engineering Department, Faculty of Agriculture and Natural Resources, Aswan University, Aswan, Egypt; 10 Department of Biotechnology, College of Science, Taif University, Taif city, Saudi Arabia; 11 Agricultural Botany Department, Faculty of Agriculture, Suez Canal University, 41522 Ismailia, Egypt

**Keywords:** *Bacillus Megaterium* BM18-2, Cadmium, GFP, Hybrid Pennisetum

## Abstract

Researchers have reported that *Bacillus megaterium* BM18-2 reduces Cd toxicity in Hybrid Pennisetum, but understanding the interaction between plants and associated endophytes is crucial for understanding phytoremediation strategies under heavy metal stress. The current study aims to monitor the colonization patterns of GFP-labeled endophytic bacteria BM18-2 on Hybrid Pennisetum grass. Additionally, it will monitor Cd’s effect on plant bacterial colonization. Confocal laser scanning microscopy of plant roots infected with gfp tagged BM18-2 revealed that the bacterium colonised root hairs and epidermal cells at the early stage of colonization, and over time, the bacteria penetrated to the internal tissues following their colonization of the stem and leaf. The roots, stems, and leaves of H. Pennisetum grown in Cd-contaminated soil contained a higher number of bacteria than those grown in normal soil. The result of Cd translocation indicated the condensation of heavy metals in the root cells and stem, while no Cd was found in the leaf. The study will also look for the enzymatic activity of bacteria BM18-2 and use Leadmium Green AM dye to track how Cd is taken up and moved through the plant. The enzymatic activity results showed that BM18-2 can produce catalase and amylase, but did not record any cellulase or lipase activity. As a result, the pattern of useful endophytic BM18-2 colonization through H. Pennisetum grass will aid in the application and maintenance of these bacteria in farming, and it presents new opportunities for the development of innovative strategies in the fields of agriculture and biotechnology.

## Introduction

Endophytic bacteria are advantageous microorganisms that inhabit the inner tissue of different plant organs, such as roots, stems, leaves, seeds, and fruits, without causing any detrimental effects (Wilson 1995; Ryan et al. 2008; Pitiwittayakul et al. 2021; Al-Hawamdeh et al. 2024). The bacteria enhance plant growth by producing plant hormones such as indole-3-acetic acid, making phosphate soluble, releasing siderophores, and providing plants with resistance against both living and non-living stressors (Gaiero et al. 2013; Etesami and Maheshwari 2018; Chen et al. 2024). Additionally, some bacterial endophytes assist plants in the biological nitrogen fixation (BNF) process, which converts nitrogen gas (N2) into usable forms like ammonium and nitrate within the host plant (Bhattacharjee et al. 2008; Santi et al. 2013; Kandel et al. 2017). As a result, incorporating bacterial endophytes into agriculture has the potential to reduce chemical fertilizers used to promote crop plant growth and quality, avoid plant pathogens, and make farming more environmentally friendly in the future.

In a heavy metal-stressed environment, the interaction between microbes and plants is essential for the adaptation and survival of both organisms. Microorganisms can employ several methods to eliminate heavy metals present in the environment (Pham 2022; Pande 2022). Beneficial interactions between endophytes and plants have gradually become the focus of many studies addressing ecological restoration, such as deserts (Jain et al. 2021) and heavy metal-contaminated lands (Liu et al. 2022). *Bacillus* (Zhou et al. 2021; Ali et al. 2022), *Enterobacter* (Ghosh et al. 2021; Wang et al. 2023), and *Pseudomonas* (Qian et al. 2022; Xing et al. 2023) are some PGPBs that could be used for bioremediation.

Bacillus species, which are common rhizobacteria that help plants grow, have a lot of potential for cleaning up contaminated soil because they are very resistant to heavy metals (Sharma 2021). Although *Bacillus* strains can survive a wide range of stress conditions, one of their most useful characteristics is their ability to form spores (Qin et al. 2022; Zocca et al. 2022). This enables their application in agriculture as PGPB inoculants (Ngalimat et al. 2021; Pham 2022). As stated by Ali et al. (2022) and Yu et al. (2023), *Bacillus* can lower the amount of exchangeable Cd in rhizosphere soil by immobilizing Cd with anionic groups on the cell wall or by securing Cd ions both inside and outside of cells. Biosorption, bioaccumulation, and bioprecipitation are the most common heavy metal removal strategies of the genus *Bacillus* (Ontañon 2018; Kumawat 2021; Khan 2022). Thus far, numerous research studies have documented the assistance of plant growth-promoting *Bacillus* spp. bacteria in plant phytoremediation (Glick 2010; Sessitsch 2013; Wang 2021). Researchers have found *Bacillus megaterium*, one of the most studied bacteria, to be common in soils contaminated with metals (Abdelkrim et al. 2018). Researchers have characterized B. megaterium’s metal tolerance for metals like Ni (Rajkumar et al. 2013), Cd (Han et al. 2020), Pb (Han et al. 2020), Cu, and Zn (Abdelkrim et al. 2018). Researchers in Portugal isolated *Bacillus megaterium* strain STB1 from the rhizosphere soil of a polluted estuarine environment. This strain can encourage the germination and early growth of many different plant species and is resistant to high salt and heavy metal concentrations. (Nascimento et al. 2019). When Cd was present, *Bacillus megaterium* BM18-2 worked well to improve the growth of Hybrid Pennisetum (Wu et al. 2019).

Green fluorescentt protein (GFP)-labeled bacteria were successfully used in several studies to follow the bacterial endophyte colonization pathways through the plant tissues and organs (Liu et al. 2006; Fan et al. 2012; Qiao et al. 2017). Liu et al. (2006) observed that the GFP-labeled *B. megaterium* (C4) could enter the maize roots through the cracks in the lateral root junction openings and then penetrate to the vascular tissues following migration to stems and leaves, while rice colonization occurred through the root tip in the zone of elongation and differentiation and the junctions between the primary and lateral roots. On the other hand, (GFP)-labeled *Paenibacillus* sp. (B1) was found to colonize root surfaces, epidermal and cortical tissue of maize (Li et al. 2017).

A little plant species has the ability to accumulate high levels of heavy metals in their tissues, which are called hyperaccumulators (Krämer 2010; Tian et al. 2017). Hybrid Pennisetum grass was produced by hybridization of *Pennisetum americanum (L.)* Leeke and *Pennisetum purpureum* Schumach and was characterized by high yield production and improved forage quality compared to the parent species (Premaratne and Premalal 2006; Li et al. 2016). In addition, the plant also showed a high resistance ability to adverse conditions such as salinity and heavy metal toxicity (Li et al. 2016; Wu et al. 2019). Therefore, exploring the heavy metal accumulation process in the different parts of plants will help in explaining the physiological and genetic variation associated with plant adaption to heavy metal stress (Alonso-Blanco et al. 2009; Krämer 2010), as well as improving plant phytoremediation strategies for toxic metals (Chaney et al. 2007).

In our previous study, *Bacillus megaterium* BM18-2 isolated from Hybrid Pennisetum showed promising ability to improve plant growth and Cd tolerance of Hybrid Pennisetum grass in pot and field experiments (Wu et al. 2019). Therefore, the present study focuses on constructing a GFP-labeled BM18-2 isolate and inoculating GFP-labeled bacteria to Hybrid Pennisetum seedlings in order to track the spread of bacterial cells in plants in the presence or absence of Cd. The purpose of these trials is to evaluate the plant-bacteria association’s potential for applicability in a metal-contaminated environment. In addition to observing Cd translocation through different parts of the plant and evaluating the bacterial ability for enzyme production.

## Materials and methods

### Construction of GFP labeled BM18-2 strain

BM18-2 was examined for antibiotic sensitivity using six different antibiotics (chloramphenicol, kanamycin, rifampicin, tetracyclines, ampicillin, and spectinomycin) by the disc diffusion method (Hudzicki 2016; Mitsuhiro et al. 2001). After screening, the two bacteria were found resist chloramphenicol at concentrations of 5–10 µg/mL. This character was considered later as a screening marker for the GFP strain.

Specific protoplast transformation methods were used to obtain the GFP-tagged strain (Barg et al. 2005; Chuping et al. 2010). BM18-2 single colony was inoculated into LB liquid media comprising of (g/L) 10 tryptone, 5 yeast extract, and 5 NaCl. The pH of the medium was adjusted to 7.0–7.2. After 24 h incubation at 37 °C, one mL of bacterial growth was transferred to 50 mL LB medium and incubated at 37 °C, 200 rpm, until late growth. The bacterial cells were collected by centrifugation at 5000 rpm, then the pellet was suspended in 10 mL of protoplast buffer with the addition of lysozyme solution (0.2 mg/mL) to the mixture and incubated at 37 °C until the bacterial liquid was clear. After that, centrifuge the solution at low speed, remove the supernatant, and leave the cells, which are protoplasts. Finally, the protoplasts were suspended in the protoplast buffer and centrifuged again. The cells were resuspended in 500 µL of protoplast buffer, and an appropriate amount of sterile glycerol was also added and dispensed in 500 µL to a 1.5 mL heart tube, stored at −70 °C.

The plasmid DNA was mixed with 100 µL of protoplasts after dissolving and placed on ice for 10–15 min. Then 300 µL of PEG-P was added, incubated for 2–3 min at room temperature, then an appropriate amount of protoplast buffer was added, mixed well, centrifuged at low speed, and removed the supernatant. The pellet was collected and resuspended in 1 mL of protoplast buffer at 28 °C for 45 min and then shaken at 200 rpm for 45 min. A 100 µL of agar overlay layer was prepared and mixed with the cell suspension, then poured into the LB plate containing the appropriate chloramphenicol antibiotic. The plate was examined for colony growth after incubation at 30 °C for 24 h.

Multiple subculture transfer was carried out to confirm the genetic stability of GFP-labeled strains obtained, and the transfer is continuously performed seven times in a week. Secondary growth 12 h (24 split growth, 30 min breeding generation), a total of 168 generations, after each transfer, through the purple appearance. Subsequently, the strain is stored in sterilized glycerol at −70 °C.

### Determination of GFP tagged BM18-2 colonization time for H. Pennisetum roots

The GFP-labeled BM18-2 strain was inoculated into LB broth shaken at 200 rpm at 30 °C for 24 h, centrifuged at 6000 rpm for 10 min, then the cell pellets were suspended in sterile distilled water and adjusted to an OD600 of 0.3. Hybrid Pennisetum seedlings at appropriate stages of growth were collected, and their roots were washed well under tap water, then immersed in a bacterial suspension (OD600 = 0.3), and incubated at 26 ± 2 °C. After that, the seedling roots were surface sterilized using sodium hypochlorite (1%, v/v) for 5 min, washed three times with sterile water, and the root samples were examined for GFP bacterial colonization at different intervals (0, 4, 10, 24, 48, and 72 h) using fluorescentt microscopy (ZEISS Axioscope 5 Upright Microscope).

### Effect of bacterial concentration on H. Pennisetum colonization during Cd stress

Two concentrations of the GFP-labeled BM18-2 strain were prepared (OD600, 0.3 and 0.6) after bacterial growth on LB broth at 200 rpm at 30 °C for 24 h. The bacterial culture was divided into two flasks, and their concentration was adjusted to OD600 (0.3 and 0.6) by diluting with LB broth. For each concentration, about 40 seedlings of H. Pennisetum were prepared and soaked for 48 h at 26 ± 2 °C, and the control seedlings were immersed in LB only. The plant seedlings were then transplanted in the pots arranged in the greenhouse. CK1, including control seedlings in soil without Cd, and CK2, for control seedlings transplanted in 50 mg/kg Cd soil, while the bacterially inoculated seedlings were divided into two groups, the first transplanted in Cd-uncontaminated soil and the other in 50 mg/kg Cd-contaminated soil. The experiment extended for 4 weeks, and the plant samples were collected every week and surface sterilized to check the bacterial colonization in root, stem, and leaf using fluorescentt microscopy (ZEISS Axioscope 5 Upright Microscope). Additionally, the bacterial counts were also determined every week. Samples from root, stem, and leaf were surface sterilized using sodium hypochlorite (1%, v/v) for 5 min, washed three times with sterile water, and then ground in a sterilized mortar. The plant extract (100 µL) was spread over LB plates and incubated at 30 °C for 24 h. Three replicates of three independent plating assays were used to determine the average colonization value.

### Monitoring Cd translocation through H. Pennisetum

The root, stem, and leaf of H. Pennisetum seedlings treated with or without 40 µM Cd for 4, 10, 24, and 48 h were stained using the Leadmium™ Green AM dye. The plant samples were immersed in 20 mM Na_2_-EDTA for 15 min at room temperature, then washed in ddH_2_O three times for 10 min each time. A stock solution of the dye was made by adding 50 µL dimethyl sulfoxide to a vial of the dye and then diluted to 1:10 with 0.85% NaCl (Zhao et al. 2013). The samples were immersed in the diluted stock solution at 37 °C for 2 h in the dark, then washed with 0.85% NaCl three times. And finally stored in the dark at 4 °C until investigation by confocal laser scanning microscopy with excitation wavelengths of 488 nm and a barrier filter of 590/50 nm.

### Determination of bacterial enzyme activity

The enzymatic activities were carried out by growing the bacterial isolates in LB broth, then after incubation for 24 h at 30 °C on a rotating shaker (200 rpm), aliquots of 100 µL were inoculated on the specific culture media for each enzyme to be investigated.

For the catalase enzyme the cultures were mixed with 3–4 drops of H_2_O_2_ in test tubes to observe the effervescence. The Amylase enzyme was detected by spreading 100 µl of bacterial culture to LB media supplemented with 1% soluble starch, and after incubation for 48 h at 30 °C, the plates were flooded with 1% iodine. Lipase was detected by streaking the bacteria on LB media supplemented with 1% of previously sterilized tween80. After incubation for 72 h at 30 °C, the plates were observed for halo zone formation. Cellulase enzyme was determined by inoculating the bacteria to Carboxylmethyl cellulose agar containing (g/l) KH_2_PO_4_ 1, MgSO_4_.7H_2_O 0.5, NaCl 0.5, FeSO_4_.7H_2_O 0.01, MnSO_4_.H_2_O 0.01, NH_4_NO_3_ 0.3, CMC 10, and agar 15. The plates were incubated at 30 °C for 5 days. After that, the agar medium was flooded with Congo red (1% w/v) and investigated for clear zone formation. The Enzymatic Index (EI) was expressed by the relationship between the average diameter of the degradation halo zone and the average diameter of the bacterial colony growth (Hankin and Anagnostakis 1975; Dogan and Taskin 2021).

### Statistical analysis

The finding data were analyzed using the IPM SPSS20 statistical program. The data were summarized using the mean, standard deviation, and one-way analysis of variance (one-way ANOVA). To identify significant differences among the data, the Duncan’s multiple range test (DMRT) was conducted.

## Results

### Construction of GFP labeled BM18-2 strain

For selecting the suitable genetic transformation technique for BM18-2. The bacterium was investigated for the presence of endogenous plasmids, and the result indicated that BM18-2 did not contain any plasmids. Therefore, three transformation methods were examined: electroporation method [1], chemical competent transformation method [2], and protoplasm for finally determining a stable transformation method.

**Fig. 1:**
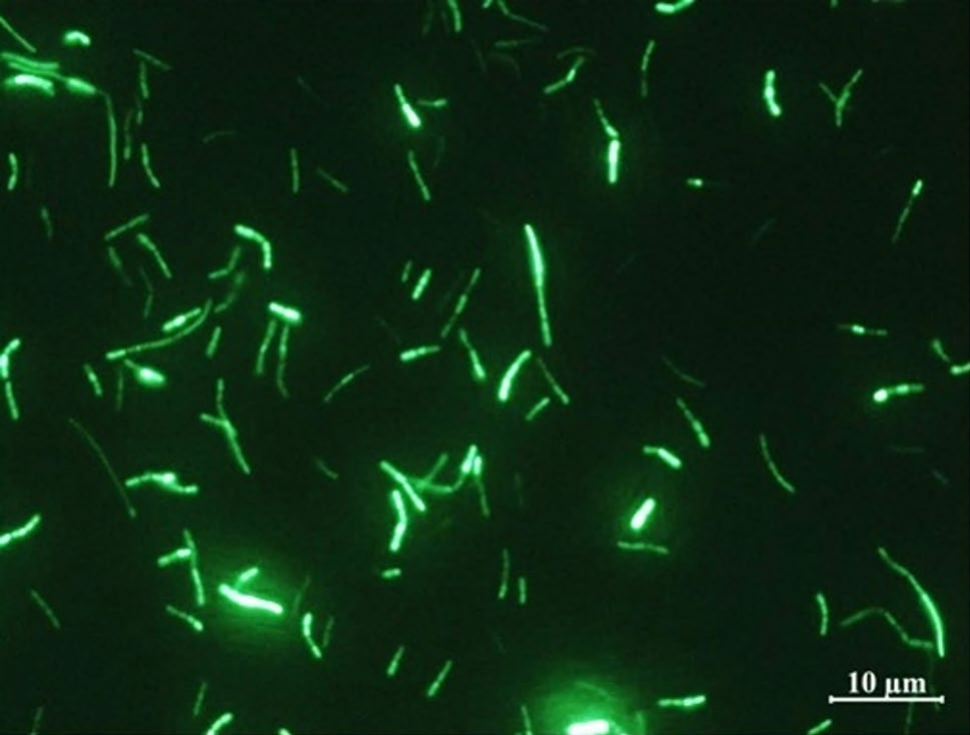
Green fluorescentt protein labeled *Bacillus megaterium* (GFP-BM18-2) prepared by the plastid transformation technique

A total of 127 transformants were picked by chemical transformation using electroporation, but there were three transformant forms, which were similar to wild-type BM18-2, and there were the same. Further verification showed that the transformants were all false positives and could not fluoresce. Consequently, the plastid transformation method, through the preparation and transformation of multiple protoplasts, was applied, and finally obtained 16 transforming marker bacteria that can fluoresce (Fig. 1).

### Determination of GFP tagged BM18-2 colonization time for H. Pennisetum root

**Fig. 2:**
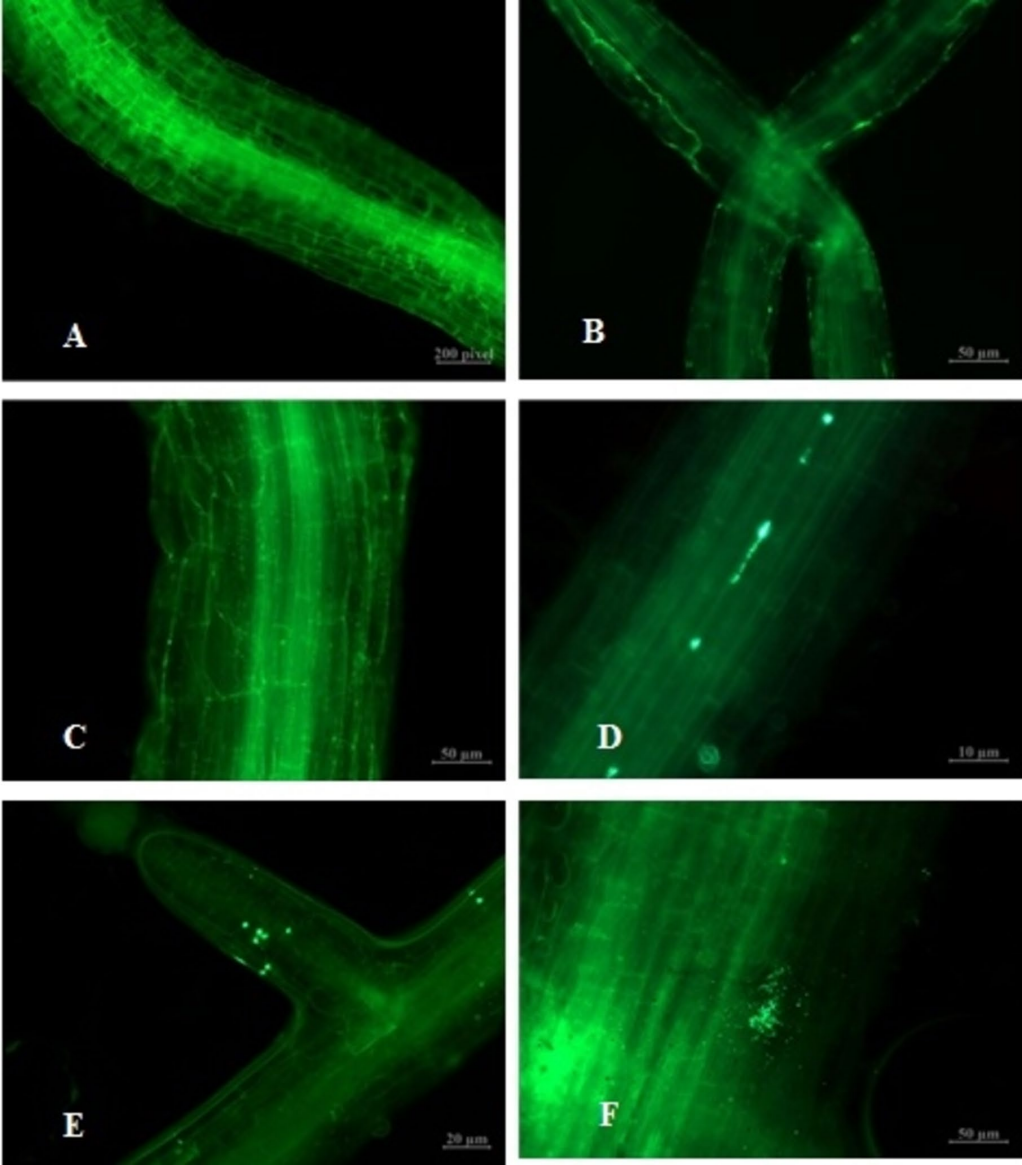
Colonization of the GFP-labeled BM18-2 bacterial cells in Hybrid Pennisetum root at different time, **A**. Control, **B**. 4 h, **C**. 10 h, **D**. 24 h, **E**. 48 h and **F**. 72 h

Colonization of the internal cells of H. Pennisetum root by GFP-labeled BM18-2 was investigated at different intervals of time. The results indicated the absence of GFP bacteria in the interior of the root cells after 4 and 10 h (Fig. 2B and C), with the bacteria only visible on the root’s outer surface. However, few bacterial cells were investigated in the root tissue after 24 h **(**Fig. 2D**)**, while several bacterial cells were observed clearly and remained for more than 48 h **(**Fig. 2E and F). Moreover, GFP bacteria displayed root hairs and epidermal cells of the seedling root at the early stage of colonization, and over time, they also appeared in the cortical cells.

### Effect of bacterial concentration on H. Pennisetum colonization during Cd stress

**Fig. 3:**
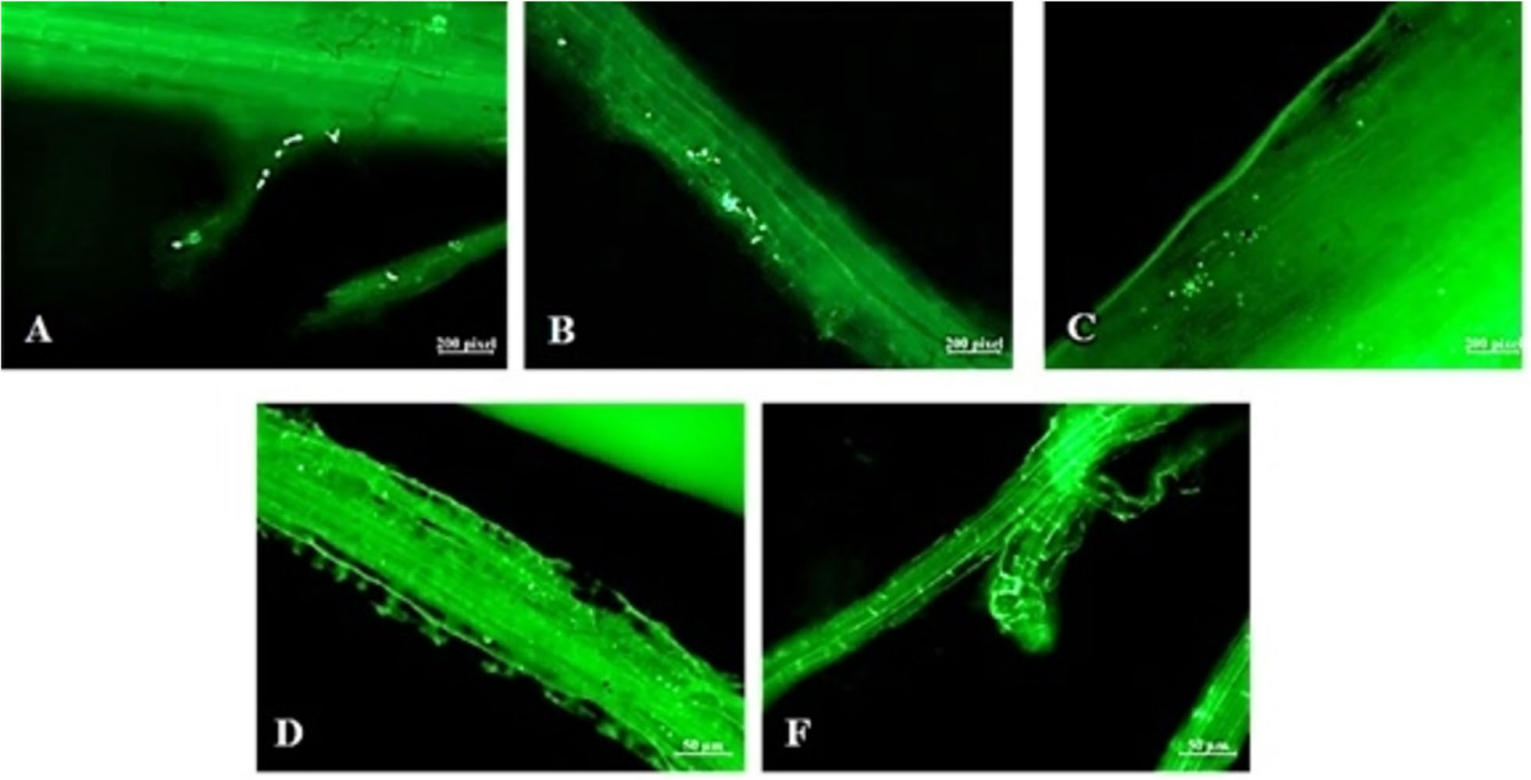
Colonization of GFP labeled BM18-2 in the root of H. Pennisetum seedlings grown in Cd free soil after **A**. 1 week, **B**. 2 week, **C**. 3 week and **D**. 4 week of bacterial inoculation, **F**. Control plant root

**Fig. 4:**
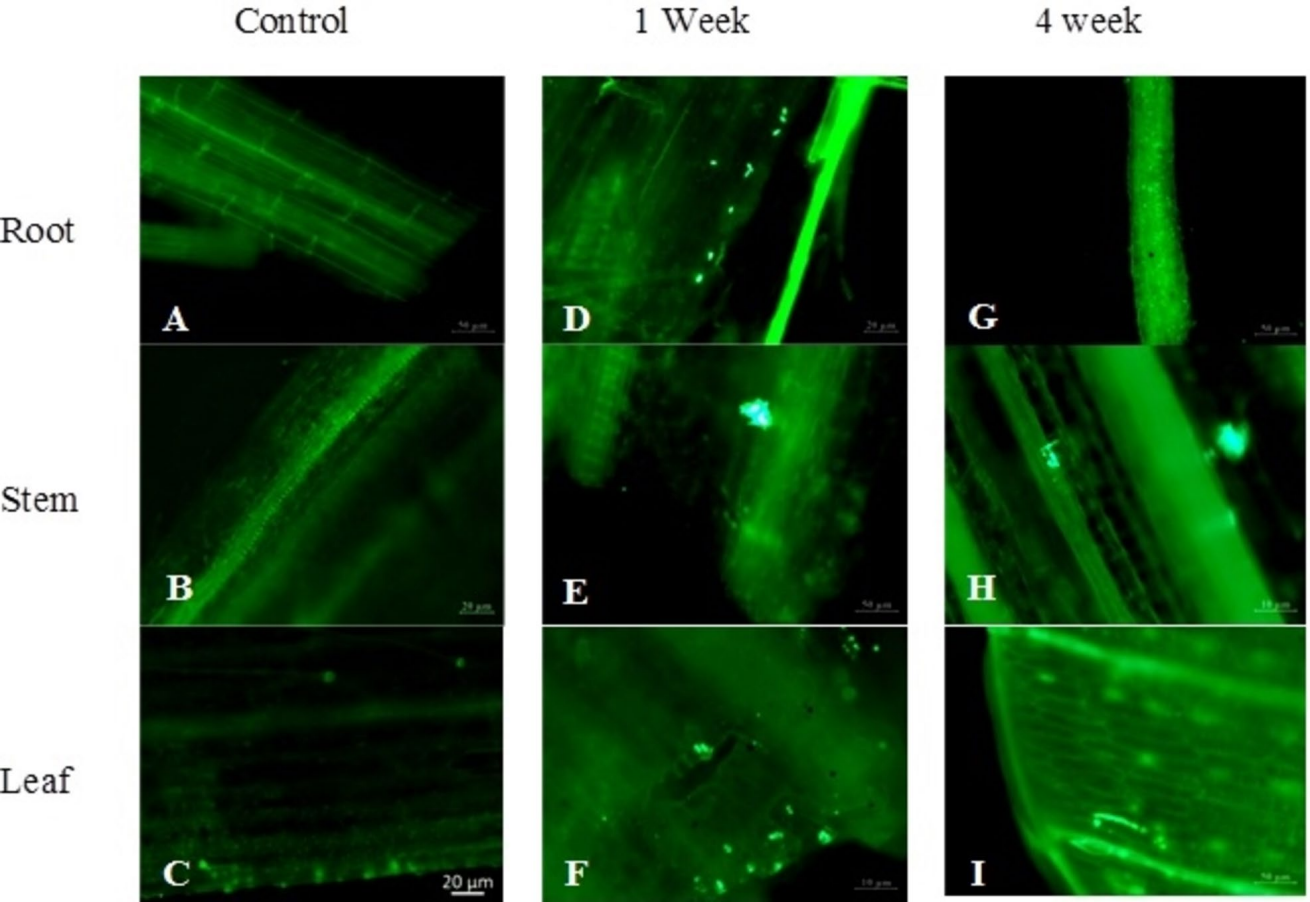
Colonization of GFP labeled BM18-2 in the root, stem and leaf of H. Pennisetum seedlings grown in Cd contaminated soil, **A**. control root, **B**. control stem, **C**. control leaf, **D**. root, **E**. stem and **F**. leaf after 1 week and **G**. root **H**. stem and **I**. leaf after 4 week of bacterial inoculation

The ability of endophytic BM18-2 to colonize H. Pennisetum seedlings on a long-term pattern was investigated in the presence and absence of Cd toxicity (Figs. 3 and 4). Figure 4 was demonstrated that the colonization in the plant roots reached the intercellular spaces and intracellular tissue, including the vascular bundles, following migration to the stem and leaf during the experimental period. The bacterial colonization inside the H. Pennisetum seedlings inoculated with OD_600_ = 0.3 of GFP was investigated. The results revealed that in the case of Cd-free soil, GFP bacteria accumulated mainly in the roots (46 × 10^2^ CFU/g FW) until the fourth week (86 × 10^2^ CFU/g FW) (Table 1), while little bacterial counts were recorded in the stem and leaf (222 and 104 × 10 CFU/g FW) after the first week and then decreased to 14 and 18 CFU/g FW at the end of the experiment. In contrast, the colonization of bacteria differed in the Cd-contaminated soil, where the high count of GFP bacterial strain recorded after the first week in the root, stem, and leaf was 67 × 10^2^, 10 × 10^2^, and 24 × 10^2^ CFU/g FW, respectively, and then decreased gradually to reach 104 and 218 CFU/g FW in the stem and leaf, respectively, while remaining high in the root at 24 × 10^2^ CFU/g FW (Table 1).

The same result was reported for the bacterial concentration OD_600_ = 0.6. The bacteria in normal soil exhibited the root cells from the first week 21 × 10^2^ CFU/g FW to the end of the trial 11 × 10^2^ CFU/g FW (Table 1). However, 248 and 131 CFU/g FW were recorded in the stem and leaf after the first week and then decreased to 8 and 7 CFU/g FW in the stem and leaf after 4 weeks (Fig. 4D). On the other hand, the behavior of bacteria differed under Cd stress, where the high count of GFP bacterial strain reported after the first week in the root, stem, and leaf was 160 × 10^2^, 14 × 10^2^, and 25 × 10^2^ CFU/g FW, respectively, and then decreased gradually to reach 84 and 14 CFU/g FW in the stem and leaf, respectively, but remained high in the root at 46 × 10^2^ CFU/g FW (Table 1).

**Table 1:** Showed the mean log of GFP labeled BM18-2 counts in root, stem and leaf of H. Pennisetum in root

Groups	1 w	2 w	3 w	4 w	P value < 0.05
Root					
A	6668.3_a_^A^	2608_b_^D^	1668.3_d_^C^	2371.0_c_^C^	< 0.001**
B	16,013_a_^D^	5000_b_^A^	2610_d_^B^	4617.6_c_^B^	< 0.001**
C	4641_b_^B^	2800_c_^C^	2713_c_^A^	8571_a_^A^	< 0.001**
D	2069_b_^C^	4151_a_^B^	109.33_d_^D^	1093_c_^D^	< 0.001**
P Value < 0.05	< 0.001**	< 0.001**	< 0.001**	< 0.001**	
Stem					
A	1024.6_a_^B^	151.3 _c_^D^	222.0_b_^A^	104.7_d_^A^	< 0.001**
B	1427.0_a_^A^	299.7_b_^B^	37.3_d_^C^	84.0_c_^B^	< 0.001**
C	222.0_a_^C^	439.0_b_^A^	10.0_d_^D^	14.7_c_^C^	< 0.001**
D	248.5 _a_^C^	189.5 _b_^C^	61.33 _c_^B^	8.3_d_^C^	< 0.001**
P Value < 0.05	< 0.001**	< 0.001**	< 0.001**	< 0.001**	
leaf					
A	2368.5_a_^B^	32.0_d_^C^	383.3_b_^A^	218.7_c_^A^	< 0.001**
B	2520.7_a_^A^	195.0_b_^A^	8.0_c_^D^	14.0_c_^B^	< 0.001**
C	1040.0_a_^C^	26.7_b_^C^	28.7_b_^C^	18.3_c_^B^	< 0.001**
D	131.5_a_^D^	86.0_b_^B^	42.7_c_^B^	7.7_d_^C^	< 0.001**
P Value < 0.05	< 0.001**	< 0.001**	< 0.001**	< 0.001**	

Different lower case letters indicate significant differences between time intervals (weeks) at p < 0.05

Different upper-case letters indicate significant differences between groups at p < 05

A grass inoculated with OD_600_ 0.3 of GFP and grown in Cd contaminated soil,

B grass inoculated with OD_600_ 0.6 of GFP and grown in Cd contaminated soil

C grass inoculated with OD_600_ 0.3 of GFP and grown in Cd free soil,

D grass inoculated with OD_600_ 0.6 of GFP and grown in Cd free soil. All data are the means of 3 independent biological replicates and are expressed as means ± standard deviation

### Monitoring Cd translocation through H. Pennisetum

**Fig. 5:**
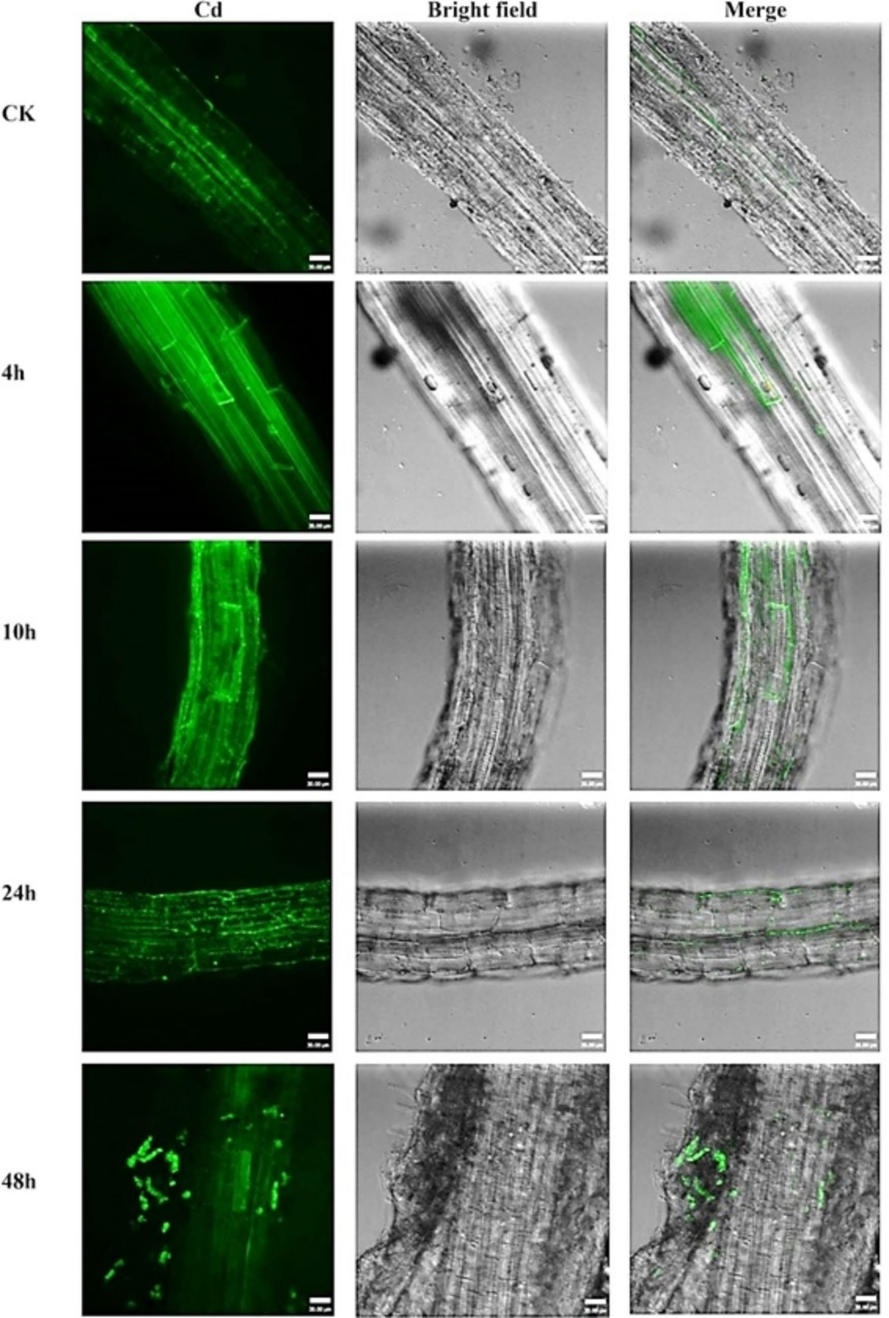
Confocal laser scanning microscope images of Hybrid Pennisetum root immersed in 40 μm of Cd after 4, 10, 24 and 48 h and treated with Leadmium™ Green AM dye. The green fluorescentce refers to association of Cd with the pigment

**Fig. 6:**
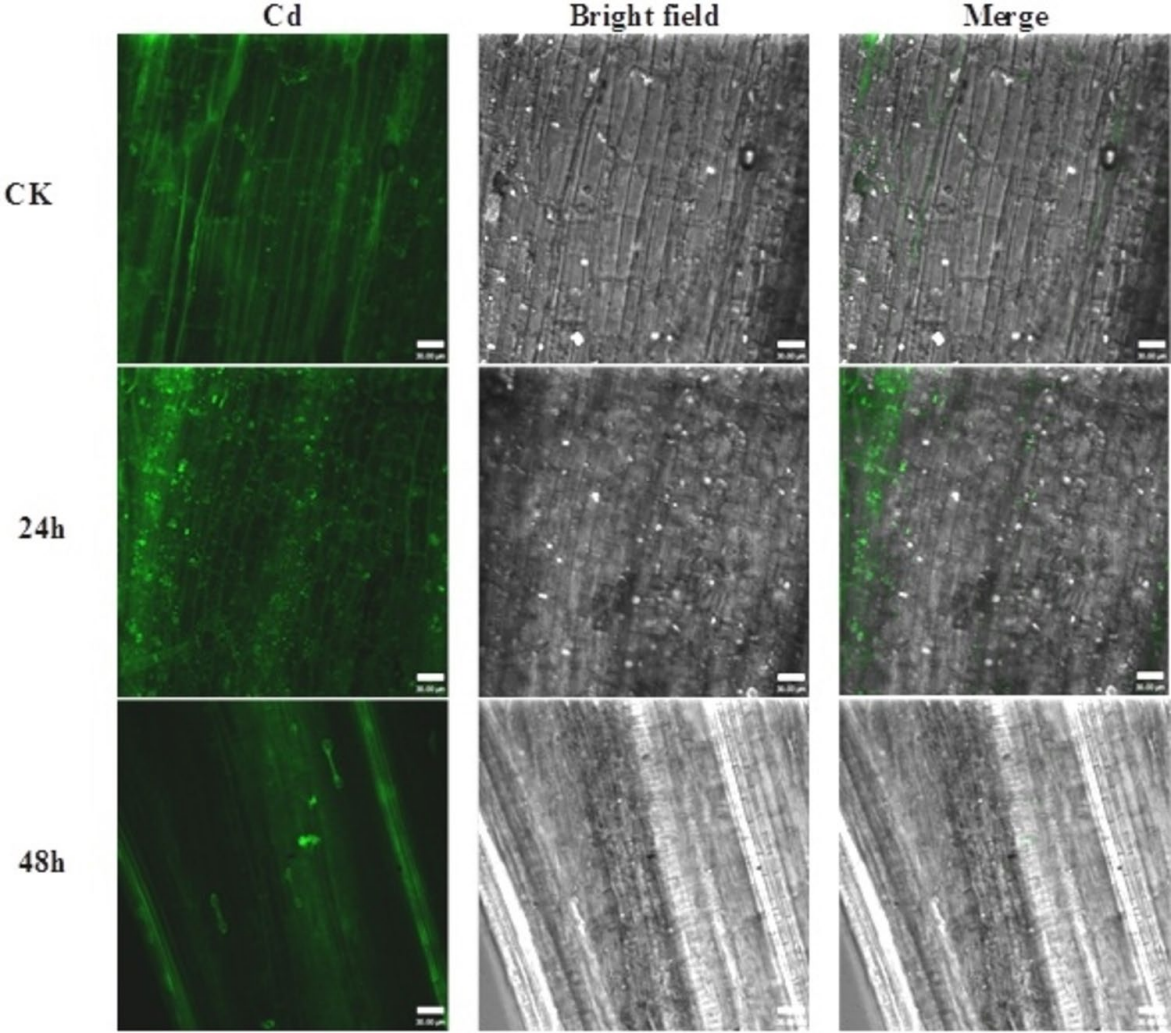
Confocal laser scanning microscope images of Hybrid Pennisetum stem immersed in 40 μm of Cd after 24 and 48 h and treated with Leadmium™ Green AM dye. The green fluorescentce refers to association of Cd with the pigment

Cd localization in the root stem and leaf of H. Pennisetum grass was investigated using Leadmium™ Green AM dye at different intervals of time (Figs. 5 and 6). At 4 h, few green colors were observed in the root, while after 10 h, the intensity of the green fluorescentt color increased gradually and was distributed through different tissues of the root until the vascular tissue at 24 h. The highest concentration of green fluorescentt color in the plant root was observed after 48 h of incubation (Fig. 5).

The green fluorescentt colors started to appear in the stem after 24 h and remain relatively with the same intensity after 48 h (Fig. 5). However, the intensity of green color in the stem is less than that detected in the root. On the other hand, the fluorescentt green color wasn’t detected in the leaf.

### Determination of bacterial enzymes production ability

The results of enzymatic detection demonstrated that the endophytic BM18-2 was able to produce amylase with a clear zone formed around the bacterial colony after addition of iodine solution over the media and resulted in an enzymatic index (EI) of about 1.04 (Table 2). In addition, the bacterial ability to hydrolyze H_2_O_2_ by catalase enzymes was reported, and bubbles were formed due to the production of oxygen (O_2_) according to the following equation:

2 H_2_O_2_ → 2 H_2_O + O_2_.

On the other hand, the bacterial isolate BM18-2 did not show ability for lipase or cellulase enzymes, where no inhibition zones were observed in their media.

**Table 2:** Determination of extracellular enzymatic activity of *Bacillus megaterium* BM18-2 *

Term	Result	Enzymatic index (EI)
Amylase	+	1.04 ± 0.04
Catalase	+	0
Lipase	−	0
Cellulase	−	0

* The results are expressed in mean ± standard deviation (n = 3)

## Discussion

Recently, numerous researchers have employed endophytic bacteria for plant growth promotion (PGP) to enhance their standing among agronomists and environmentalists by reducing chemical exposure into the environment (Li et al. 2016). A grass that once supported a well-known population of endophytic microorganisms will most likely pass on the microorganisms to its progeny along with any potential benefits (Ferreira 2008). In this study, Hybrid Pennisetum seedlings were successfully colonized by BM 18 − 2, which had been tagged with a GFP. The purpose of the GFP technology and microscopic analysis was to facilitate the simple identification of BM 18 − 2 colonization in the root, leaf and stem in a natural setting. The endophytic BM18-2 showed the ability to colonize plant roots and was clearly observed under the microscope after 24 h of inoculation. Although the distribution of bacterial cells was not uniform along the different root cells, it was reported that the majority of bacterial cells exhibited root hairs and epidermal cells, which may be because this region being rich in exudates (Fan et al. 2012). Liu et al. (2006) also detected that the GFP-labeled *B. megaterium* C4 directly penetrated the cortex of the wheat root through the epidermis in the root hair zone, and a similar mechanism can be used by the bacteria to colonize rice and maize. Liu et al. 2006 recorded the presence of *Bacillus megaterium* C4 cells mainly in the intracellular spaces in maize and rice. Zhang et al. 2011 demonstrated that B. subtilis N11 colonized the lateral roots of banana plant roots. The Hybrid Pennisetum roots colonized the four different endophytic bacterial strains, including *Bacillus*, *Enterobacter*, *Pantoea*, and *Sphingomonas* (Li et al. 2016). Moreover, GFP-labeled *Paenibacillus* sp. B1 could successfully colonize the surface and the epidermis cells of maize roots (Li et al. 2017).

After that, the BM18-2 cells were also observed in the cortical tissue. Endophytes invade the cortex, quickly pierce the epidermal rupture point, and then spread to vascular tissue. (Byregowda et al. 2022). The bacterial colonization through H. Pennisetum in the long run also reached stems and leaves, but most were still in the roots. Similar findings were found by Hallmann and Berg (2006) who demonstrated that *Klebsiella aerogenes* HGG15/gfp, the endophytic bacterial population decreased in above-ground tissues, such as stems and leaves, compared to roots. It was proven that one of the plant responses to metal stress is to change root structure, such as increased surface area, due to the formation of root hairs and lateral roots, which provide more active sites to be colonized by useful bacteria (Vitti et al. 2014).

The present results revealed that the colonization of endophytic BM18-2 in the H. Pennisetum grass was also affected by Cd stress, where high counts of bacteria were recorded in the root, stem, and leaf compared to the non-stress condition. This may be considered as a bacterial response mechanism against the unfavorable condition. Also, the growth of the plant inoculated with BM18-2 was greatly improved in soil contaminated with Cd compared to the non-inoculated plant, which reflects the plant growth promotion ability of endophytic BM18-2 in addition to increasing plant resistance to Cd toxicity. It was found that beneficial bacteria, under stress conditions such as salinity, drought, heavy metals, lack of a nutrient, etc., can exert a positive effect on the plant compared to non-stress conditions (Etesami and Maheshwari 2018).

Bacterial isolate BM18-2 exhibited amylase and catalase activity by producing clear halo zones and bubbles on respective agar plates. Under Cd stress, increased antioxidant levels have been observed in rice treated with different microorganisms, such as *Bacillus cereus* S6D1–105 (Jabeen et al. 2022). The establishment of internal colonization by endophytes relies on the surface properties of the bacteria and the enzymes that degrade plant polymers. The synthesis of cellulases, chitinases, proteases, and pectinases is significant (Carro and Menendez 2020). The extracellular enzymatic activities of endophytic bacteria also provide plants with protein and polysaccharide, which in turn improve plant nutrition (Choi et al. 2005). In the study conducted by Bhatt and Vyas (2014), rhizobacterial (PGPR) strains were isolated from the rhizospheric soils of plants growing in semi-arid regions, and the results showed that 27% of bacterial isolates produced lipase and 53% produced amylase.

## Conclusion

This study sheds light on the beneficial interactions between *Bacillus megaterium* BM18-2 and Hybrid Pennisetum under cadmium (Cd) stress, highlighting its implications for sustainable agriculture. The study successfully traced colonization pathways from root hairs to internal tissues using GFP-labeled bacteria, highlighting the bacterium’s adaptability and its function in promoting plant growth by reducing Cd toxicity. The BM18-2 strain demonstrated encouraging characteristics that promote plant growth, such as catalase and amylase activities, which are critical for plant resilience. Furthermore, the strain’s ability to restrict Cd translocation predominantly to root tissues underscores its potential to diminish Cd accumulation in edible plant parts. This redistribution not only safeguards the aerial portions of the plant, which are essential for biomass and quality, but also establishes BM18-2 as an intriguing option for phytoremediation strategies that aim to contain heavy metals in contaminated soils. The cumulative findings of this study position B. megaterium BM18-2 as a stress-resistant and versatile PGPB for biotechnological and agricultural applications. It is recommended that additional research be conducted on molecular pathways to enhance our understanding of the absorption and transport of Cd in plants, thereby facilitating the development of advanced phytoremediation strategies. Such studies could open the path for the creation of advanced microbial consortia and bioinoculants that maximize phytoremediation attempts and improve crop resilience in soils contaminated with heavy metals.

## Data Availability

All data are presented within the article.
